# Chitosan Film as Eco-Friendly and Recyclable Bio-Adsorbent to Remove/Recover Diclofenac, Ketoprofen, and Their Mixture from Wastewater

**DOI:** 10.3390/biom9100571

**Published:** 2019-10-05

**Authors:** Vito Rizzi, Fabio Romanazzi, Jennifer Gubitosa, Paola Fini, Roberto Romita, Angela Agostiano, Andrea Petrella, Pinalysa Cosma

**Affiliations:** 1 Università degli Studi “Aldo Moro” di Bari, Dip. Chimica, Via Orabona, 4-70126 Bari, Italy; vito.rizzi@uniba.it (V.R.); fabioromanazzi94@gmail.com (F.R.); roberto.romita@uniba.it (R.R.); angela.agostiano@uniba.it (A.A.); 2 Consiglio Nazionale delle Ricerche CNR-IPCF, UOS Bari, Via Orabona, 4-70126 Bari, Italy; j.gubitosa@ba.ipcf.cnr.it (J.G.); p.fini@ba.ipcf.cnr.it (P.F.); 3 Dipartimento di Ingegneria Civile, Ambientale, del Territorio, Edile e Chimica, Politecnico di Bari, via Orabona, 4-70126 Bari, Italy; andrea.petrella@poliba.it

**Keywords:** chitosan film, emerging pollutants, diclofenac, photodegradation, adsorption

## Abstract

This paper reported the first example on the use of chitosan films, without further modification, to remove and recover, through bio-sorption processes, the emerging pollutant Diclofenac from water. The latter was adopted as a model, among non-steroidal anti-inflammatory drugs, by obtaining a maximum adsorption capacity, q_max_, on chitosan of about 10 mg/g, under the applied experimental conditions of work. The literature gap about the use of chitosan films, which was already used for dyes and heavy metals removal, to adsorb emerging pollutants from water was covered, claiming the wide range application of chitosan films to remove a different class of pollutants. Several parameters affecting the Diclofenac adsorption process, such as the pH and ionic strength of solutions containing Diclofenac, the amount of the bio-sorbent and pollutant, and the temperature values, were investigated. The kinetics and the adsorption isotherms, along with the thermodynamic parameters (ΔG°, ΔH°, and ΔS°) were also evaluated. The process occurred very efficiently, and Chitosan/Diclofenac amounts dependent, remove about the 90% of the pollutant, in 2 h, from the tested solutions, through electrostatic interaction involving the carboxylic moiety of Diclofenac and Chitosan amino groups. This finding was confirmed by the pH and salt effects on the bio-sorption process, including swelling measurements of Chitosan films and by FTIR-ATR analysis. In detail, the maximum adsorption was observed at pH 5, when pollutant and Chitosan were negatively and positively charged, respectively. By reducing or increasing the pH around this value, a reduced affinity was observed. Accordingly, the presence of salts retarded the Diclofenac removal screening its charges, which hinders the interaction with Chitosan. The sorption was spontaneous (ΔG° < 0) and endothermic (ΔH° > 0) following the pseudo-second order kinetic model. The process was Diclofenac and Chitosan amount dependent. In addition, the Freundlich and Temkin isotherms well described the process, which showed the heterogeneous character of the process. Experiments of the complete desorption were also performed by using NaCl solutions 0.25 M (like sea water salt concentration) proposing the reuse of the pollutant and the recycling of the bio-sorbent lowering the associated costs. The versatility of the adsorbent was reported by exploring the possibility to induce the Diclofenac light-induced degradation after the adsorption and by-products adsorption onto chitosan films. To emphasize the chitosan capacity of treating water, the removal of another pollutant such as Ketoprofen and the mixture of Diclofenac and Ketoprofen were investigated. In this way, a green and eco-friendly production-pollution prevention technology for removing emerging pollutants from water was presented, which reduced the overall environmental impact. This illustrated experiments both in static and dynamic conditions for potential industrial applications.

## 1. Introduction

With a growing world population, in recent years, the human consumption of pharmaceuticals has increased, which is leading to an increased concentration of pharmaceutical compounds in wastewater [1]. Unfortunately, most of them are persistent in conventional wastewater treatment plants and, then, they are released in the treated water for human need, as tap water. This represents risk for human health [1,2]. Specifically, medicines are considered a worrying class of emerging pollutants (EPs) since they induce a physiological response affecting both non-target individuals and species [3]. Regarding this concern, although there are no legal discharge limits for these pollutants, some regulations have been published, and the Directive 2000/60/EC was the first mark in the European water policy, which established a strategy to describe how to prioritize high-risk substances. Among pharmaceutical compounds, Diclofenac ((2-(2,6-dichlorophenylamino) phenylacetic acid, DCF) commonly used as analgesic, anti-arthritic, and antirheumatic agent, is still one of the most frequently detected pharmaceuticals in the water environment [2]. Not surprisingly, DCF, together with three antibiotics (azithromycin, clarithromycin, and erythromycin) and three estrogens (E1, E2, and EE2), have included, by the EU Decision 2015/495, in the first watch list of substances to be monitored in all member states to collect high quality monitoring data of emerging pollutants in the European water bodies. Coelho De Escobar et al. [4] recently have shown that the 15% of DCF was excreted unchanged after consumption and it has been detected in both the influents and effluents of wastewater treatment plants at high concentrations [4]. Therefore, strategies must be developed for reducing the amounts of this Non-Steroidal Anti-Inflammatory Drug (NSAID) in water, as of all other EPs. In this sense, Lin et al. [5] reported the use of Advanced Oxidation Processes (AOPs), for the DCF removal, could be considered as a powerful tool for this purpose, even though a by-product derived from AOPs may exhibit even higher toxicity compared to the original pollutant [5]. Furthermore, the heterogeneous photocatalytic oxidations processes show, as a whole, low selectivity to the target contaminants, and the photocatalysts cannot differentiate highly toxic target pollutants from other organic compounds of low toxicity [6]. Therefore, adsorption processes are still preferred and considered more useful for removing EPs. In this case, DCF, from water, thanks to their simplicity, has low initial costs, and by-product-free operations [2,5]. For this purpose, different adsorbent materials can be used, i.e., chelating or ion-exchange resins, mineral supports, active carbon, and residues from chemical industry, agriculture, or fishing [7]. However, considering the biosorption processes, in the optic of greener and sustainable applications, the sorbent should have a biological origin, and, among the bio-adsorbents, chitosan is one of the most used in this field [8,9]. This amino-polysaccharide exhibits interesting properties among biopolymers due to its cationic character in acidic solutions, which is ascribable to protonated amino moieties (pK_a_ value of ~ 6.0–7.0) [7,10,11]. The electrostatic interactions with anionic molecules are usually favored, with chitosan positively charged amino groups interacting with negatively charged species [7]. Rizzi et al. [8,12] studied the treatment of industrial effluents for cleaning water from industrial dyestuffs. The influence of chitosan on filtration performances for heavy metals removal was presented by Lam et al. [13] by highlighting the recent interest toward chitosan as wide-ranging adsorbent material [13,14]. Starting from these considerations, about the removal of pollutants from water, the use of bio adsorbents [15,16,17,18,19,20] is so contemplated and, with the particular regard to DCF, to our knowledge, only few papers have been reported in literature, by mainly discussing the use of chitosan-based systems [15,16,17,18,19]. Chitosan, which is a by-product from the alkaline deacetylation process of chitin, is an amino polysaccharide known for its distinctive properties, besides biodegradability and biocompatibilities [8].

In this work, the use of chitosan is proposed as chitosan films (CH), for the removal of DCF from water, by obtaining q_max_ values of approximately 10 mg·g^−1^. The important aspect of this paper is related to the possibility of regenerating the adsorbent by means of solutions containing NaCl. The film appeared as a thin membrane that can be placed in and easily removed [8,12]. Therefore, the possibility to perform consecutive cycles of adsorption and adsorption/desorption is explored, which opens novel horizons in the use of chitosan films by extending their applications, and lowering the associated costs by reusing both the adsorbent and the pollutant itself. Another aspect preliminarily analyzed in the present paper was the possibility to work both under static and dynamic conditions, by proposing in flux experiments for a potential scaling-up for industrial applications. High adsorption efficiencies, which removes around 80% of DCF, in a few minutes, were obtained. Lastly, as an alternative, the possibility to induce the solid state DCF photo-degradation after the adsorption onto CH was investigated. Since the DCF recover was almost complete, the degradation was also accomplished in solution after the desorption in 0.25 M NaCl. About this, for the first time based on our knowledge, the DCF by-products, induced by light irradiation, were also high efficiently removed from water, which further emphasizes the CH potentialities. Moreover, the adsorbent exhibited high efficiency in the removal of another NSAID, i.e., Ketoprofen (Kp), from water with a potential application in the removal of the mixture Kp and DCF. Kp is also frequently found in surface waters, which constitutes a potential risk for aquatic ecosystems [21,22,23,24]. Besides the studies reported in literature about its removal [25,26,27,28,29,30], the proposed use of CH to treat water from the Kp, according to our knowledge, has been not yet investigated. Therefore, regarding this aspect, to better emphasize the capacity of CH, preliminary results are discussed in this paper, which opens a novel possibility to treat water polluted with emerging pollutants.

## 2. Results and Discussion

### 2.1. Effect of Adsorbent Dosage and DCF Concentration

**Figure 1:**
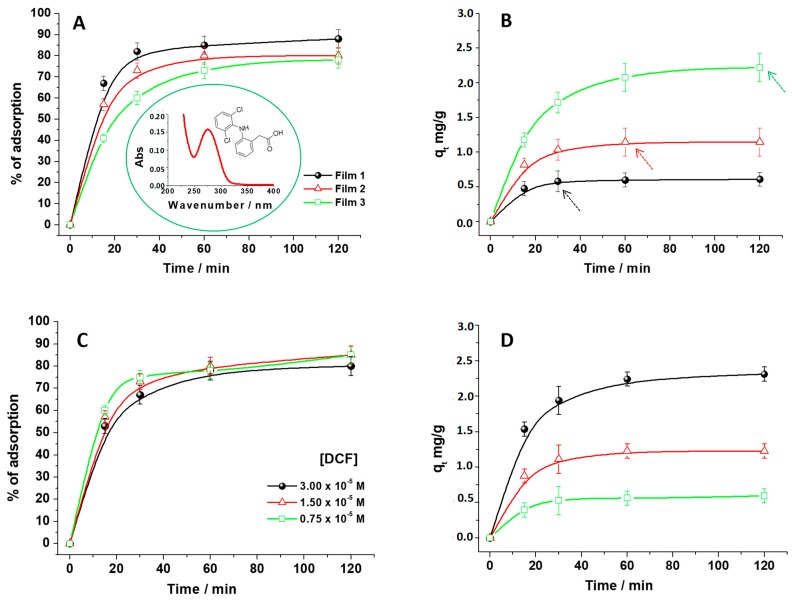
(**A**,**B**) The percentage of DCF adsorption onto chitosan film, with the related adsorption capacities, q_t_, by adopting a DCF solution 1.50 × 10^−5^ M, at pH 5, and three different chitosan film weights, Film 1 > 2 > 3, (**C**,**D**) by changing the DCF concentrations, 0.75 × 10^−5^ M, 1.50 × 10^−5^ M, 3.00 × 10^−5^ M, at pH 5, using Film 2.

During this work, the DCF removal from water was investigated by monitoring its UV-Vis absorption spectrum in aqueous solutions, which are purposely contaminated with the pollutant. In particular, the DCF main signal at 277 nm (inset in Figure 1A), due to a π–π transition [22], was used as a diagnostic to infer the DCF adsorption percentage in the presence of CH. At first, three CH with different masses were used, with Film 1 > 2 > 3 (Figure 1A) in a 1.50 × 10^−5^ M (5 mg·L^−1^) DCF solution at a pH of 5 (the DCF solution and pH after the adsorbent addition).

Considering the first minutes of contact time, the adsorbent amount effect that occurred was more evident: at 30 min, the following percentage of DCF removal were obtained: 85%, 70%, and 60% for Film 1, 2, and 3, respectively. Furthermore, the amount of the adsorbed DCF increased when the contact time was increasing, which reached a plateau after 120 min. This is a condition in which the removal was quite complete. Overall, this finding suggests that increasing the CH weight more, free sites were available on the adsorbents for hosting DCF [8,20,21]. In Figure 1B, the influence of the chitosan amount on DCF adsorption was carefully explored by calculating the q_t_ values (Equation (1)). On one hand, by increasing the adsorbent amount, the relative adsorption of DCF molecules increased (the plateau region beginning, indicated with arrows, was more quickly reached in the presence of Film 1). On the other hand, the adsorption capacity decreased. This result, which is in agreement with Reference [21], can be explained by considering that, using a large amount of adsorbent, the adsorption sites partially remained unsaturated during the adsorption process. This reduces the q_t_ values as a whole. In particular, observing Figure 1B, at the beginning of the adsorption process, the presence of a large quantity of free sites for the DCF adsorption increased the q_t_ values. However, extending the contact time, the free sites decreased and reduced the relative DCF adsorption, which reached a plateau. This behavior could be attributed to the presence of repulsive forces between DCF molecules in solution and those adsorbed, which hindered the NSAID diffusion into the adsorbent structure, leveling off the q_t_ values and the adsorbed DCF %. This was observed by Rizzi et al. [21], in a similar study regarding dyestuff adsorption onto a hydrogel adsorbent.

The role of the free active sites was confirmed by changing the amount of DCF (3.00 × 10^−5^ M, 1.50 × 10^−5^ M, and 0.75 × 10^−5^ M) fixing the chitosan weight (Film 2), as shown in Figure 1C,D. The DCF adsorption percentage showed variations in the first minute of contact between Film 2 and DCF solutions (Figure 1C), which indicates that diluting the DCF solutions, the pollutant removal increased. However, important changes can be better discovered by calculating the associated q_t_ values (Figure 1D). The relative maximum adsorption capacities were quickly obtained, after a few minutes, by employing an NSAID small amount (see the first point in which the plateau region was observed), since, by reducing the ratio DCF/adsorbent, more adsorption sites were available [20]. The maximum adsorption capacity, q_max_, was experimentally inferred and it was about 10 mg·g^−1^. More information about the adsorption process were obtained by studying the diffusion role after applying the Weber-Morris model. In particular, the intraparticle diffusion model used for this purpose was: q_t_ = k_int_ × *t*^1/2^ + C, where C represents the thickness of the boundary layer and k_int_ is the kinetic constant related to the intra-particle diffusion rate in mg/g·min^1/2^ [5]. When the intraparticle diffusion is the limiting stage of the adsorption, the graph of q_t_ versus *t*^1/2^ is a straight line passing through the origin [23]. By changing the amount of chitosan films, from Film 1 to 3 (Figure S1A), multiple linear segments were necessary to fit the experimental data, which suggests that two or more steps may be involved during the DCF adsorption process [5,23]. The same was observed by changing the DCF concentrations (Figure S1B). This finding suggested that, under our experimental conditions, the DCF adsorption process was constituted by two sequential phenomena. At first, the diffusion from the solution to the external surface of the adsorbent, followed by a second step corresponding to the intra-particles’ adsorption and diffusion. Indeed, the experimental points, at each condition of work, can be separated into two groups (Figure S1A,B). The former represents the first stage, while the latter one is referred to the second stage [23]. As suggested by Lin et al. [5], during this second stage, since the DCF concentration decreased, the adsorption process slowed down and approached an equilibrium state, which is considered as the third stage. However, the regression line of the second stage did not pass through the origin of the plot for all the investigated conditions, which suggests that the intra-particle diffusion was not the rate limiting step [5,23].

### 2.2. Effect of pH

**Figure 2:**
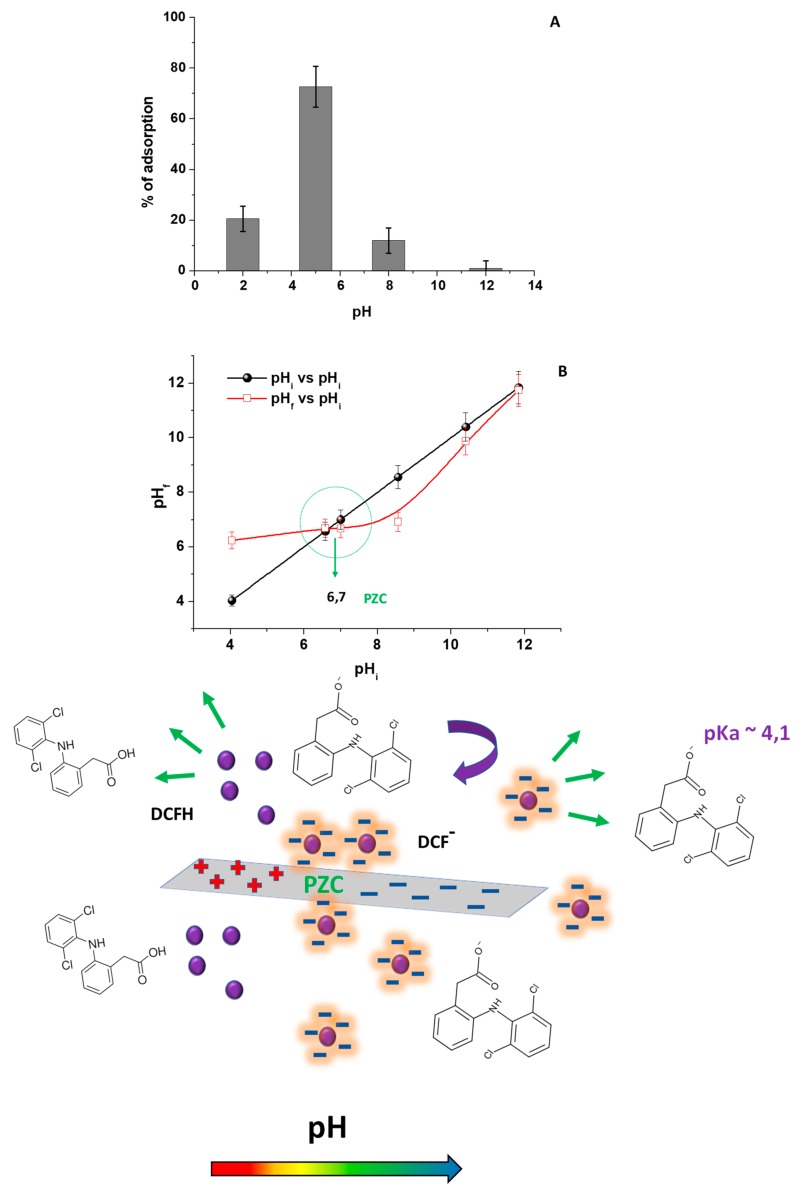
(**A**) The percentage of DCF adsorption (considering 120 min as contact time) onto chitosan film (Film 2) at several pH values (from a DCF solution 1.50 × 10^−5^ M). (**B**) The determination of the pH_PZC_ of the chitosan film by using the drift method. The cartoon reported in the figure depicts the nature of interaction between DCF and CH, by changing the pH values. The violet spheres represent the DCF molecules.

For studying the pH effect during the DCF adsorption onto CH, DCF solutions with pH values ranging from 2 to 12 were investigated by adding HCl or NaOH, according to the case. For avoiding changes of pH values during the adsorption process, due to the presence of acetic acid added during the chitosan film preparation, the used adsorbent (Film 2) was previously neutralized with NaOH and washed several times with fresh water. As reported in Figure 2A, the adsorbed DCF percentage on chitosan, calculated at each pH values, increased from pH 2 to 5. This is a condition in which the maximum adsorption occurred, and decreased after this pH value, which shows a DCF removal is not significant at pH 12. To better understand this behavior, the CH point of zero charge (PZC) was experimentally determined by the drift method (Figure 2B) [21]. In agreement with literature, the observed cross section region of the curves in Figure 2B, indicated that the pH_PZC_ of CH was around pH 7. It means that, below pH 7, the chitosan amino groups are positively charged, which tends to significantly deprotonate toward the pH_PZC_. After this pH value, chitosan becomes mainly negatively charged [24]. Moreover, in accordance with the carboxylic moiety presented in the DCF chemical structure (Scheme S1), the NSAID pK_a_ is reported to be 4.15 [25]. Then, below this pH value, the DCF is present as neutral DCFH (protonated form of DCF), while, after this value, as anionic DCF (DCF^−^).

Starting from these considerations, the results reported in Figure 2A can be rationalized. In detail, below pH 5, DCF was largely present as DCFH and CH and showed a positively charged surface due to the protonation of amino groups. As a result, a slight affinity between DCFH and the adsorbent was observed with a scarce contribution of electrostatic interactions. Specifically, at pH 2, DCF was protonated by the excess of H^+^ in water, which reduces the pollutant/adsorbent electrostatic attraction [31]. At pH 5, DCF was mainly present as DCF^−^ and, at the same time, chitosan was positively charged. Then, the electrostatic interactions between the carboxylic moieties of DCF^−^ and the chitosan amino groups positively charged take place, which favor the DCF^−^ removal. At pH > 5, i.e., pH 8, DCF continues to be DCF^−^, while the chitosan amino groups were mainly deprotonated and reduce the affinity between the adsorbent and DCF^−^, which suggested an electrostatic repulsion between the DCF and CH negative charges [7,16,18,19]. The cartoon in Figure 2, graphically describes the hypothesized adsorption mechanism.

### 2.3. FTIR-ATR Measurements

**Figure 3:**
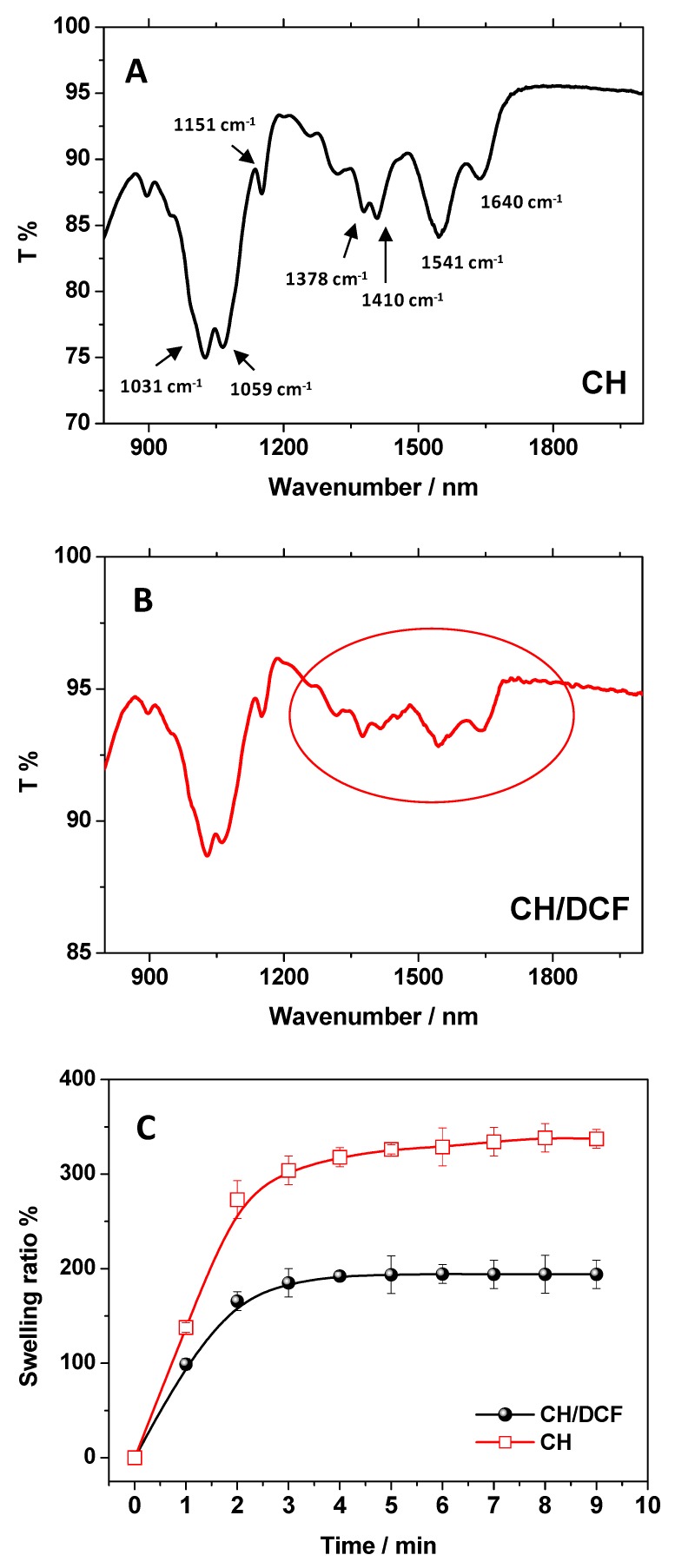
FTIR-ATR measurements (detailed wavenumber region 600–2000 cm^−1^) of (**A**) CH, (**B**) CH/DCF, and (**C**) swelling ratio measurements of CH and CH/DCF.

In order to confirm the hypothesized adsorption mechanism, involving electrostatic interaction, FTIR-ATR measurements of CH were performed both before and after the DCF adsorption (Figure 3A,B). As a first step, in Figure 3A, the typical CH spectrum in absence of DCF is reported. Focusing the attention in the wavenumber’s region 600–2000 cm^−1^, in our experimental conditions, the characteristic CH IR bands were distinguished. The amide I band (-NHR-CO- stretching) was detected at 1640 cm^−1^ and, at 1541 cm^−1^, the overlapping of the signal relative to the chitosan NH_2_ bending (amide II) with the carboxylate stretching vibrations of acetate anions was observed. At 1410 cm^−1^ and 1378 cm^−1^ the contribute of acetate anions and C-N bond were also detected, respectively. The C-O-C asymmetric and symmetric stretching were observed at 1151 cm^−1^ and 1059 cm^−1^, respectively, and at 1031 cm^−1^ is located the C-O vibration of the alcoholic moieties [10,11].

As a second step, to evidence the effect of DCF on the chitosan vibration modes, the FTIR-ATR spectrum of CH after the DCF adsorption was collected, and the obtained results are reported in Figure 3B. 

After the DCF adsorption, the FTIR-ATR spectrum of CH/DCF system significantly changes (see the black circle in Figure 3B). In details, the amide I and II signals changed, reversing their ratio, indicating the involvement of amino groups during the interaction with the adsorbate [10]. At the same time, the C-N signal shifted from 1378 cm^−1^ to 1370 cm^−1^, confirming this finding. Further, the signals in the 1200–1500 cm^−1^ and 900–1100 cm^−1^ ranges were subject to slight shifts of wavenumbers, indicating a hydrogen bonding reorganization. More specifically, the signal at 1410 cm^−1^ reduced its relative intensity and, at the same time, the C-O-C vibration modes slightly changed [10,19]. These observations, in agreement with results obtained during the pH study, suggest the presence of electrostatic interaction between DCF and CH during the adsorption process. 

Moreover, the swelling ratio measurement of the CH before and after the DCF adsorption resulted in agreement with these results (Figure 3C). It is worth to mention that the CH swelling ability (Equation (2)) in water is well known, and it was related to the protonation degree of chitosan amino groups. In particular, the CH film, when swollen in water, increased its mass of more of 300% during the first minutes. In presence of the adsorbed DCF, the swelling was of 200%, suggesting a more hydrophobic character of the adsorbent after the DCF load, attributable to the screening of positive charges present on the surface of chitosan mediated by the anionic DCF [10]. To strengthen the role of the electrostatic interaction, involving DCF^−^ anion and the positively charged chitosan, adsorption experiments were performed also varying the solution ionic strength by adding electrolytes [17,18].

### 2.4. Effect of Salts in DCF Solutions

**Figure 4:**
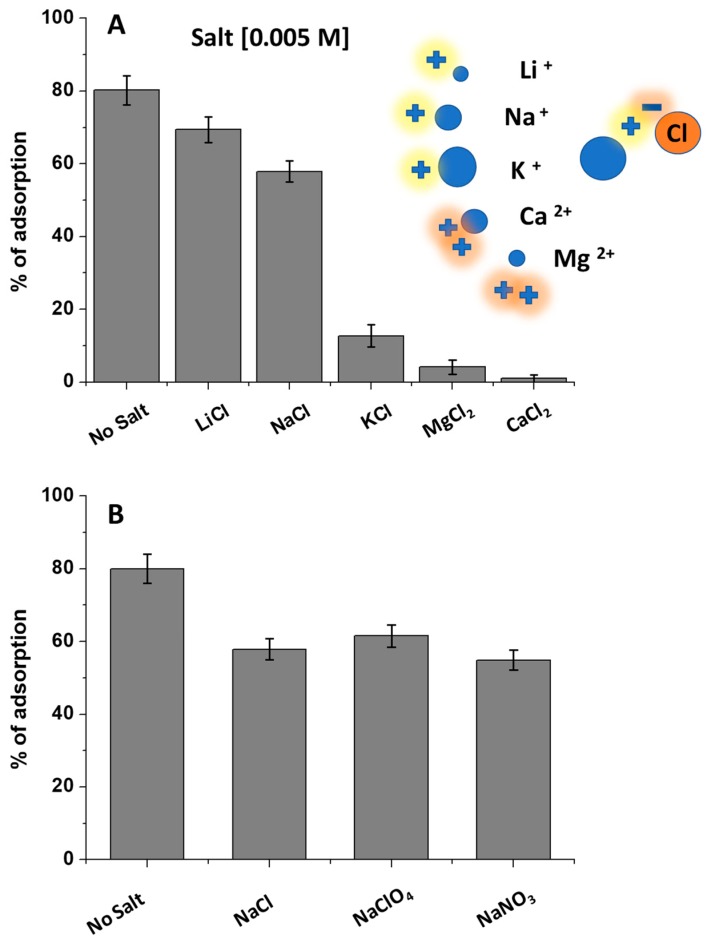
(**A**) Percentage of DCF adsorption onto chitosan film (Film 2), at pH 5, (from a DCF solution 1.50 × 10^−5^ M), in the presence of different salts, and (**B**) by changing the nature of the cation and anion. The adopted contact time was 120 min.

To strengthen the role of the electrostatic interaction, involving DCF^−^ anion and the positively charged chitosan, adsorption experiments were performed by varying the solution ionic strength with the addition of electrolytes [17,18]. NaCl was selected as model, and different concentrations were explored by maintaining a constant DCF concentration (1.50 × 10^−5^ M), solution pH (pH = 5), and Film 2. The DCF adsorption percentage was calculated after 120 min of contact time (Figure S2). Changing the salt concentration from 0.001 M to 0.5 M, the DCF removal percentage decreased from 80%, in the absence of salt, to 60% with NaCl 0.005 M, with the complete lack of adsorption in the presence of NaCl 0.5 M. By choosing 0.005 M as a salt reference concentration, the electrolyte nature changed. In particular, fixing the anion nature (Cl^−^), the cation was changed exploring the following series: Li^+^, Na^+^, K^+^, Mg^2+^, and Ca^2+^. As reported in Figure 4A, increasing the cation size, from Li^+^ to K^+^, the DCF removal efficiency decreased and, by changing the cation associated charge, using Ca^2+^ and Mg^2+^, the effect occurred more markedly. These results suggested a cations-mediated shielding effect of the DCF negative charge, which highlights, again, the involvement of the DCF carboxylic moieties in the adsorption onto chitosan film [17,18]. Conversely, by changing the anion nature, and choosing Na^+^ as cation, the results reported in Figure 4B were obtained. The absence of significant changes in the percentage of adsorbed DCF indicated the scarce role of the inorganic anions in the adsorption. In fact, if the anion nature affects the DCF/CH interaction, it would prevent the adsorption and shield the chitosan positive charge onto the film surface, since both DCF^−^ molecules and the anions should diffuse on the surface of the film. Instead, the results indicated that the negative charge diffusion inside the film did not occur or happened slowly. This suggests a competition between DCF^−^ molecules and inorganic anions for the positive charges of the CH surface, with DCF^−^ favored compared with the inorganic anions, due to the involvement of DCF/CH interactions that are not purely electrostatic. Furthermore, this implies that the cation effect of shielding the DCF negative charge occurred directly in solution and not in the solid-state chitosan film.

### 2.5. Consecutive Cycles of Adsorption

**Figure 5:**
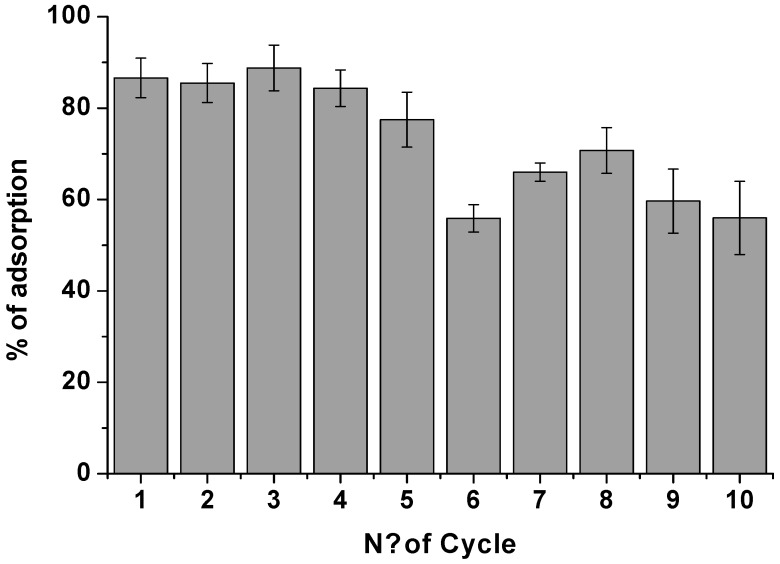
The percentage of DCF adsorption onto chitosan film (Film 2), at pH 5, (from a DCF solution 1.50 × 10^−5^ M), calculated for 10 consecutive cycles of adsorption by using the same film and by changing the DCF solution after each cycle. The contact time for each cycle was 120 min.

For highlighting the high performance of the proposed adsorbent, consecutive cycles of adsorption were performed. By selecting 120 min as contact time, a 1.50 × 10^−5^ M DCF solution at pH 5 was placed in contact with the adsorbent (Film 2) and, after the complete adsorption, (80% of DCF), the same film was used again to adsorb DCF from another fresh solution at the same concentration. The DCF adsorption percentages reported in Figure 5 and Table S1 were obtained. For the first five cycles, the NSAID removal was quite efficient, while, from the fifth to the tenth cycle, the DCF adsorption efficiency changed, passing from ~80% to a mean value of 60%. The maximum adsorption capacity of this film typology was evaluated at around 10 mg·g^−1^. Therefore, by calculating the total amount of DCF adsorbed during the 10 cycles, ~10 mg of DCF per gram of adsorbent were adsorbed, which indicates the active sites’ saturation within the tenth cycle.

### 2.6. Thermodynamic Analysis

**Figure 6:**
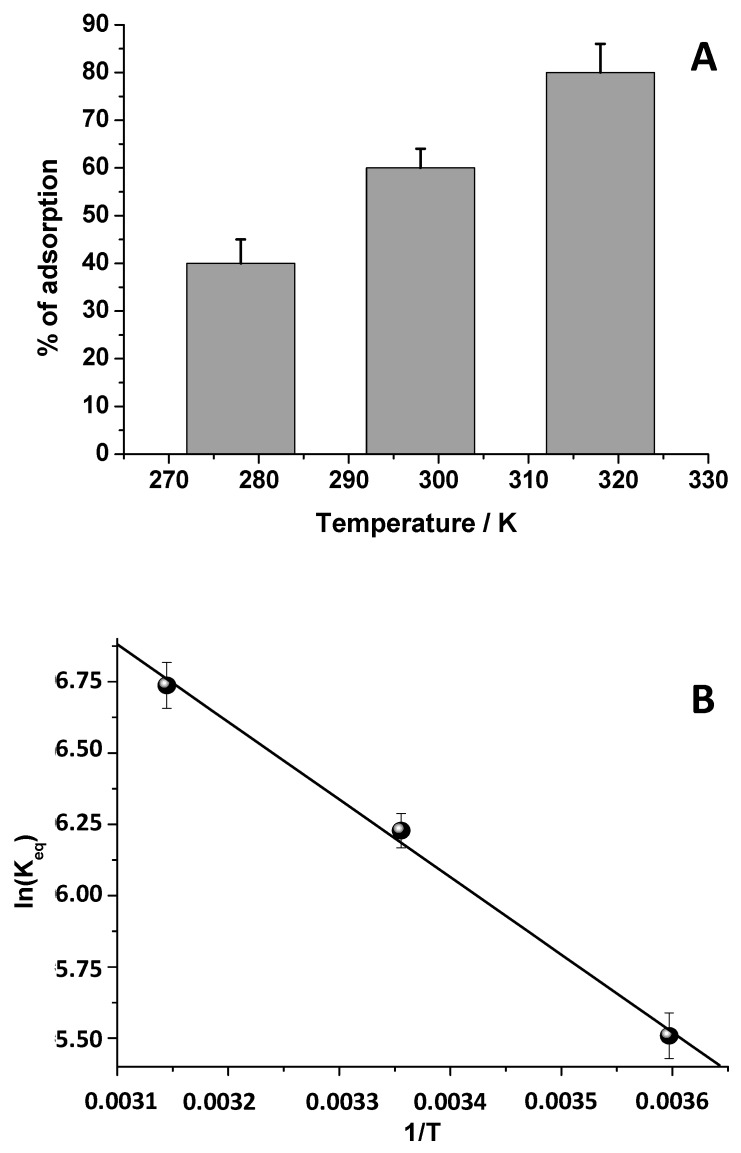
(**A**) DCF adsorbed the percentage at different temperature values (adopting as contact time 120 min). (**B**) lnK_eq_ vs 1/T. In particular, the K_eq_ represents the equilibrium constant at the selected temperature value.

Temperature is an important parameter when the adsorption processes are studied. It can increase or decrease the adsorption rate in relation to the investigated process. The temperature effect gives indication on the exothermic or endothermic nature of the adsorption process. More specifically, if the adsorption process is endothermic, when increasing the temperature, the adsorption capacity increases [29]. Thus, the influence of the operating temperature on the adsorption was studied in this paper by inferring information about the entropy and free energy. For this purpose, the DCF adsorption process was investigated by adopting three temperature values, i.e., 278, 298, and 298 K, by using Film 2 and a 1.50 × 10^−5^ M DCF solution at a pH of 5. The results are reported in Figure 6A. Clearly, by increasing the temperature values, the equilibrium DCF adsorption percentage increased, which changes from 40% to 80%. This suggests the endothermic character of the process [5].

To obtain the thermodynamic parameters, the K_eq_ values were calculated at each temperature and, by using Equations (3) and (4), the correspondent ΔG° values were inferred (Table S2). The negative ΔG° values indicated the spontaneity of the DCF adsorption process onto chitosan. Furthermore, by plotting ln K_eq_ versus 1/T (Figure 6B) and by applying Equation (5), ΔH° and ΔS° were also calculated and the results are reported in Table S2. In agreement with literature [23], the positive values of ΔH° (+22.62 KJ/mol) and ΔS° (+127.37 J/mol·K) confirmed the endothermic character of the process and the increased randomness at the adsorbent-adsorbate interface, respectively.

### 2.7. Isotherms of Adsorption

The adsorption isotherms study is an important key feature for describing the adsorption processes, with the aim of better describing the biosorption process [11]. For this purpose, the Langmuir, Freundlich, and Temkin models (Equations (6)–(8)), which are usually proposed in literature for similar studies [21,23], were used in their linearized form and, the results are reported in Figure S3. Analyzing the obtained R^2^ values, associated to each isotherm models during the data fitting (Table S3), the applicability of both Freundlich and Temkin isotherms was considered. In detail, the DCF adsorption occurred onto heterogeneous surfaces (Freundlich) and the heat of adsorption during the process linearly decreases with the coverage, due to adsorbent-adsorbate interactions (Temkin). Lin et al. [5] suggested that both mono-layer and multiple-layer adsorptions should be taken into consideration, involving both physisorption and chemisorption during the process. Furthermore, the *n* value (see Table S3), calculated by applying the Freundlich model (Equation (6)), indicated that the adsorption process was favored. Additionally, *n* represents the adsorption strength, and values ranging from 1 to 10 confirm this finding [21].

### 2.8. Kinetic Analysis

To gain more information about the dynamic of the adsorption process, the kinetic analysis was performed, by applying the pseudo-first (Equation (9)) and pseudo-second (Equation (10)) order kinetic models [20,21,23]. The analysis was carried out both by changing the DCF concentration, fixing the amount of the adsorbent, and vice versa (Figures S4 and S5). Therefore, from the q_t_ values reported in Figure 1B,D, the calculated kinetic parameters in Tables S4 and S5 indicated that the R^2^ values relative to the pseudo-second order model (R^2^ ≈ 0.99) were slightly better [23]. Besides the R^2^ values of the linear fitting, the comparison between the experimental adsorption capacities at the equilibrium, q_e,exp_, (contact time 120 min) and those obtained by applying the kinetic equations, q_e,calc_ (calculated adsorption capacities), were evaluated [21,23]. The comparison in Tables S4 and S5 clearly confirms that the application of the pseudo-second order equation better interpreted the experimental data, which confirms the key role of both the DCF and chitosan amount in the adsorption process, since the rate-controlling step depends on the physicochemical interactions between the DCF and the adsorbent [23].

### 2.9. Release of DCF and Reuse of The Adsorbent

**Figure 7:**
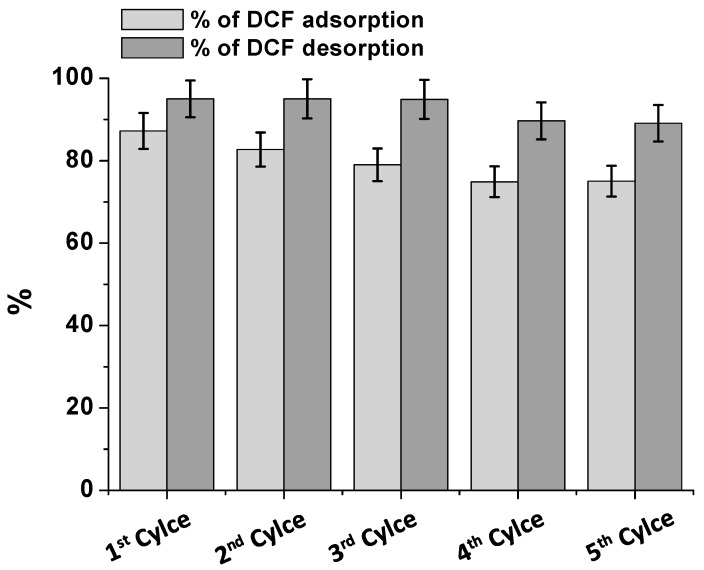
Percentage of DCF adsorption/desorption (in 0.25 M NaCl) on/from CH for five cycles. The contact time for each cycle was 120 min.

For exploring the possibility of adsorbent/adsorbate recycling, experiments of DCF desorption were also performed. In particular, due to the involvement of DCF/chitosan electrostatic interaction, and for reducing the environmental impact, the use of electrolytic solutions is proposed. As a green application, to verify the salt-effect, among the studied salts (see Figure 4A), KCl, NaCl, and MgCl_2_ solutions were selected and compared, while maintaining all experimental conditions constant. After the DCF adsorption, the chitosan film was immersed, under continuous stirring, in 0.005 M KCl, NaCl and MgCl_2_ aqueous solutions. Once again, the DCF UV-Vis absorption spectrum, collected at 30 and 60 min, was used for monitoring the DCF release. The desorbed DCF percentage, normalized for the adsorbed amount, was calculated and reported in Figure S6. The obtained results indicated that extending the contact time from 30 to 60 min, the percentage of the desorbed DCF increased, and the effect was leveled off among the proposed salts. The following results were obtained: 20% at 30 min and 50% at 60 min for NaCl, 45% at 30 min and 55% at 60 min for KCl, and 50% at 30 min and 60% at 60 min for MgCl_2_. However, to develop a more sustainable design, aiming to reduce the overall environmental impact, NaCl and KCl solutions were considered and further investigated, by exploring different salt concentrations (Figure S7). For both salts, the electrolyte concentration was changed from 0.005 M to 0.5 M and the desorbed DCF percentage was evaluated at 30 and 60 min. Some differences between NaCl and KCl can be observed only at the lowest concentrations of the salts, while, from 0.1M on, the salts effect appeared to be the same. Furthermore, there were no significative differences between 30 and 60 min. Therefore, always considering a more sustainable procedure for a cleaner production-pollution prevention, the use of 0.25 M NaCl was suggested for the DCF removal using 30 min as contact time for desorption. In this sense, the sea water could be used for this purpose, considering, as reported by Takagi et al. [26], that the salt concentration in sea water was around 0.5 M. On this ground, several cycles of adsorption/desorption were performed and the percentage of the adsorbed/desorbed DCF, for each cycle, is reported in Figure 7. Once again, the desorbed DCF percentage is normalized with respect to the correspondent adsorbed DCF. After each cycle, the adsorbent was washed with fresh water, and, subsequently, immersed in fresh DCF solutions. Clearly, if after the first cycle, the DCF adsorption slightly decreased. On the other hand, the desorbed DCF percentage was almost complete after five cycles. The obtained results suggested the possibility of reducing the procedure-associated cost and the amount of secondary pollutants potentially released into the environment, by proposing not only the possible DCF reuse, but, more importantly, the adsorbent recycle for cleaner and greener production technologies.

### 2.10. Preliminary in Flux Measurements

When in flux experiments were considered, the adsorbent was first swollen inside a syringe-like column with fresh water. Then, by a peristaltic pump, a 1.50 × 10^−5^ M DCF solution (15 mL) was made to flow through the adsorbent (Figure S8A). Therefore, after the DCF flow through the column, the eluted solution was collected and spectrophotometrically analyzed. After only 15 min, the DCF adsorption of ~80% was observed (Figure S8B), which highlights, with this preliminary experiment, that the proposed adsorbent can be used under dynamic conditions of work. The DCF in-flux desorption was also attempted by using, in this case, concentrated NaCl solutions, but the results were not so satisfactory (data not shown).

### 2.11. Photodegradation of DCF

In order to present a more advantageous approach, beside the proposed results about the DCF reuse and the adsorbent recycle, the possibility to induce photodegradation of the adsorbed DCF in a solid-state was explored during this work. The process could be used in synergy with the adsorption process for destroying the DCF removed from water. Furthermore, the adsorption of the photoinduced DCF by-products was evaluated to better show the great performance of CH in the adsorption of pollutants from water. The DCF photodegradation is well known since many works are discussed in the literature [27,28,29,30,31]. The work of Iovino et al. [30] described and reported the main oxidation steps during the UV light-induced DCF degradation, which confirmed the presence of a carbazole derivate in the first minutes of the reaction.

Therefore, the DCF photodegradation was attempted, by irradiating the chitosan film after the DCF adsorption with UV light and/or a sun simulator lamp. As a first step, a spectrophotometric investigation was performed, by monitoring the UV-Vis spectrum evolution of a simple DCF solution under irradiation (Figure S9A). After 60 min of irradiation, the collected DCF absorption spectra occurred identically both by using UV and a sun light simulator. The obtained spectra agreed well with results in literature [30], which confirms the presence of a carbazole derivative, whose main absorption contribute is around 340 nm. Starting from these results, the DCF-solid state degradation was attempted. In detail, the loaded adsorbent was placed in 30 mL of fresh water and irradiated for 1 h. Subsequently, for investigating the occurred DCF degradation, the film was dipped in an NaCl solution 0.25M for inducing the DCF release, and the UV-Vis spectrum was collected and reported in Figure S10. The typical absorption spectrum of the DCF by-products arisen from UV degradation was observed (see Figure S9A). However, in this case, the absorbance intensity at 290 nm of the UV-Vis spectrum increased, which is in agreement with the results shown by Zhang et al. [28]. This indicates different pathways of reaction followed by the DCF during the sunlight irradiation, ascribable to its solid-state degradation [29]. On the other hand, if the irradiation of the chitosan film was performed in dry conditions, by avoiding the use of water during the experiment, the DCF photodegradation does not occur. This indicates that the adsorbent can also be used not only for removing the NSAID from water, but also to preserve it from light, being this molecule is highly photo-instable.

If the DCF degradation was performed after the DCF release and the irradiation time was extended to 300 min, the complete degradation of the DCF by-products absorbing in the UV-Vis spectral region was also observed (Figure S9B). Therefore, the use of this chitosan film, as adsorbent, enabled different pathways to be followed, according to the necessity. Moreover, due to the high DCF photo-instability, it is possible that by-products from its photodegradation are also present in water. Therefore, preliminary experiments of adsorption of these substances were attempted. After the DCF degradation, adopting 60 min as contact time, the UV-Vis spectrum reported as time 0 was obtained. Therefore, the chitosan adsorbent was swollen in that solution and the related spectra were acquired at several contact time periods (Figure S11). The results indicated the high ability of chitosan not only in adsorbing DCF, but also the related by-products. After 360 min, the absorption bands were slightly visible if compared with the condition at time 0, which confirms our hypotheses (Figure S11).

### 2.12. Preliminary Experiments about the Removal of Ketoprofen, Diclofenac, and Their Mixture

In order to verify the adsorption performance of the proposed adsorbent in an EPs mixture, the removal of another EP, such as ketoprofen (Kp) from water, was also proposed (Figure S12A). Kp as DCF belongs to the family of NSAIDs and exhibits a chemical molecular structure similar to DCF (see Figure S12A) with differences related to the absence of the chlorine atoms on one of the benzene rings and the presence of the carbonyl moiety replacing the -NH- functionality between the aromatic rings [32]. The removal of this emerging pollutant is also an actual problem [32] and very interesting results were obtained during this work.

For evaluating the Kp adsorption, the Kp UV-Vis spectrum (with the main absorption band centered at 260 nm) was used, at the same experimental condition of DCF (Figure S12A). In detail, after 15 min of contact time, about 60% of Kp was removed, and this efficiency was increased to 80% after 60 min. Comparing these results with those obtained in the case of DCF (Figure 1A), it clearly arises that the NSAIDs behaved, as a whole, in a similar way, which shows high affinity toward chitosan. Therefore, the EPs mixture was studied, which demonstrated that the adsorption process was not affected by the presence of other pollutants. In particular, a mixture of Kp and DCF, at equal concentrations (1.50 × 10^−5^ M), was characterized in the presence of CH, by the UV-Vis absorption spectroscopy (Figure S12B,C): Kp exhibited an absorption spectrum similar to DCF, with an hyperchromic effect of the main absorption signal (Figure S12B). The investigated EPs mixture showed the spectrum reported in Figure S12C, with the main absorption band at 264 nm. Considering the adsorption onto chitosan film, the results (Figure S13A) indicated the high performance of the proposed adsorbent. Furthermore, in the same experimental condition applied for the DCF release, some desorption experiments were also carried out both after the single Kp adsorption and the DCF/Kp mixture. Focusing more specifically on the mixture behavior, the results (Figure S13B) indicated that, by fixing the adsorption contact time at 30 min, approximately 60% of the EPs was removed from water. Moreover, by observing the collected UV-Vis spectrum of the mixture after 30 min in contact with chitosan, it is evident that the main peak wavelength did not change, which suggests that the Kp/DCF ratio remained constant in solution after the adsorption, and the two NSAIDs were adsorbed with the same efficiency. When the desorption was investigated, and the adsorbent was dipped inside an NaCl solution 0.25 M for 30 min, the UV-Vis spectrum (Figure S13B) showed a wavelength shift of the main absorption band at 268 nm. This is indicative of the DCF preferential release with regard to Kp, where the DCF was characterized by the main absorption band at 277 nm. On this ground, without doubt, the use of chitosan opens a novel horizon in the treatment of water contaminated from EPs.

### 2.13. Comparison with Other Adsorbents

Considering different adsorbent materials [2,3,5,20,21], the recycle efficiencies that were around 100%, in 30 min of contact time, increases the adsorbent lifetime. Nonetheless, the adsorption capacity was 10 mg·g^−1^, that value can be potentially increased considering the cycles of adsorption/desorption, which lowers the process associated costs. Regarding the latter concern, for example, interesting results were obtained in the work of Antunes et al. [23], which proposed the DCF removal from aqueous solution by another bio adsorbent as Isabel grape bagasse [20,23]. In particular, besides the obtained high adsorption capacities, i.e., 76.98 mg·g^−1^, the DCF desorption was investigated by applying continuous stirring of the system after it reached equilibrium. Nonetheless, regarding the higher adsorption capacity, the authors observed that the drug was completely desorbed from the grape bagasse after 72 h of contact time. Therefore, compared with our work, a very long contact time was required. Moreover, the authors showed, as the bagasse was not only reached in cellulose and lignin, but also the presence of phenols is contemplated that could be, potentially, released in the environment lowering, as a whole, the green aspect of the process [20,23,33,34]. Another example is the use of a zeolitic imidazole framework functionalized with cetyltrimethylammonium bromide studied by Lin et al. [5] for DCF removal, with adsorption capacities from 43.95 mg·g^−1^ to 65.58 mg·g^−1^ increasing the temperature. In this case, the adsorbent was regenerated by washing with a concentrated NaCl solution. However, the synthesis of the adsorbent was elaborate and recycling for many cycles was not explored [5], if compared with the present work. Furthermore, in previous works in which chitosan was used, elaborate synthetic steps are described for obtaining chitosan adsorbents [15,16,17,18].

Hence, as a whole, the work conditions used in our study for the formation of CH can be considered safe and green since cleaner production technologies [8,12,13,14,15,16,17,18,19,20,35] decrease the environmental impact by reusing the adsorbent itself and the pollutant.

## 3. Materials and Methods

### 3.1. Chemicals

The used chemicals were of an analytical grade and samples were prepared using deionized water. Commercial grade chitosan powder (from crab shells, highly viscous, with deacetylation degree ≥75%), acetic acid (99.9%), and glycerol (+99.5%) were purchased from Sigma-Aldrich (Milan, Italy). Chitosan ^1^H-NMR (700MHz) and FTIR-ATR results were agreed, which gives us a deacetylation degree around 70%, and confirms the Sigma Aldrich manufacturer′s specification. The same commercial source was adopted for Sodium Diclofenac (C_14_H_10_Cl_2_NNaO_2_, M.W. 318.13 g·mol^−1^) and Sodium Ketoprofen (C_16_H_13_NaO_3_, M.W. 276.267 g·mol^−1^), used without further purification. A DCF stock solution with a concentration of 10 mg·L^−1^ was prepared. The pH of the various aqueous solutions, when necessary, was adjusted using diluted HCl and NaOH solutions. Dilutions were performed using deionized water.

### 3.2. Preparation of Chitosan Films

Chitosan powder was dissolved in 0.8% (*v*/*v*) aqueous acetic acid solution to obtain a 1% (*w*/*v*) chitosan concentration by constant continuous stirring for 24 h. A total of 200 μL of glycerol were added every 100 mL of chitosan acetic solution. Then, the solution was filtered through a coarse sintered glass filter and degassed for 1 h. After degassing, the chitosan solution was poured into a plastic Petri plate. This plate was maintained in an oven at 60 °C for 24 h [8,10,11,12].

### 3.3. In Batch Equilibrium Experiments

The CH adsorption capacities, with respect to DCF, were calculated in terms of *q_t_* (mg·g^−1^) at time *t*, by applying Equation (1) below [20].

(1) $$q_{t}=\frac{C_{0}-C_{t}}{W}\times V$$

where *V* represents the DCF solution volume (herein 15 mL), *W* is the dried chitosan adsorbent mass (g), and *C*_0_ and *C_t_* (mg·L^−1^) represent the DCF concentration at initial and *t* time.

More specifically, a CH fixed amount (0.05 g) into flasks containing 15 mL of DCF solution at different initial concentrations (0.75 × 10^−5^ M, 1.50 × 10^−5^ M and 3.0 × 10^−5^ M, corresponding to 2.5 mg·L^−1^, 5.0 mg·L^−1^ and 10.0 mg·L^−1^ of DCF, respectively) was used. The adsorption was performed under continuous stirring at 250 rpm, and UV-Vis absorption spectra were recorded to evaluate the DCF removal efficiency and the chitosan adsorption capacities from water. The adsorbent amount effect was also explored, changing its mass from 0.025 g to 0.10 g (0.1 g: Film 1, 0.05 g: Film 2, 0.0125 g: Film 3). In this case, the DCF concentration was settled at 1.50 × 10^−5^ M. The effect of both solution ionic strength, (by using different salts, LiCl, KCl, NaCl, CaCl_2_ and MgCl_2_, at several concentrations), and changes in pH values (ranging from 2 to 12), on the adsorption process, were also studied.

### 3.4. In Batch Desorption Experiments

After the DCF adsorption from water, 0.25 M NaCl was selected, in the optic of a green economy and cleaner production, since the best salts are able to induce the release of the adsorbed DCF. With the same approach adopted for the DCF adsorption, the UV-Vis investigation was used to assess the amount of the desorbed pollutant. In detail, after the DCF adsorption, the adsorbent was washed with fresh water to remove the not adsorbed DCF and swollen in the NaCl solution for the release. The effect of the contact time was evaluated, and 30 min occurred to be suitable for the 100% DCF recovery. Experiments were performed in triplicated and the mean values were calculated reporting the correspondent standard deviations.

### 3.5. In Flow Adsorption/Desorption Experiments

The adsorbent was placed inside a commercial syringe-like column 3.00 × 5.00 cm and then was swollen with water. Subsequently, the DCF solution (1.50 × 10^−5^ M) slowly flowed through the column, with a peristaltic pump working at 3.00 mL·min^−1^. The eluting solution was collected and analyzed by UV-Vis absorption spectroscopy.

### 3.6. Determination of Chitosan Film Zero-Point Charge

The zero-point charge pH (pH_ZPC_) of the chitosan adsorbent was evaluated by using the pH drift method [21]. A total of 30 mL of NaCl solution with a concentration of 5.0 × 10^−2^ M were utilized at different pH values ranging from 2 to 12 (pH_i_). Concentrated HCl and NaOH solutions were used for this purpose. The pH_i_ values of these solutions were measured and, subsequently, 0.025 g of adsorbent were swollen inside. These solutions were stirred at 298 K for 48 h. The final pH (pH_F_) values were measured. By reporting the pH_i_ versus pH_F_ values, the value of pH_ZPC_ was inferred at the cross section of the latter curve with the line of pH_i_ versus pH_i_.

### 3.7. Photodegradation of DCF

Among several ways to induce the oxidation of the pollutant, the photodegradation of DCF was accomplished using UV and artificial sun light. After the DCF adsorption, the adsorbent was washed with fresh water in order to remove the un-adsorbed DCF and swollen in 15 mL of water. A UV lamp (UV fluorescent lamp, Spectroline, Model CNF 280C/FE, λ = 254 nm, light flux 0.2 mW/cm^2^; Spectronics Corporation, Westbury, NY, USA) or a sun simulator lamp Model 6684 (a Xenon lamp (150 W) with a E0: 1482 mW/cm^2^, around 1.48 suns by Oriel Corporation, Stratford, CT, USA) were used to irradiate the adsorbent at several contact times. Measurements were performed by irradiating the DCF solution after the release in NaCl 0.25 M.

### 3.8. UV-Visible Measurements

UV-Vis spectra were recorded using a Varian CARY 5 UV-Vis-NIR spectrophotometer (Varian Inc., now Agilent Technologies Inc., Santa Clara, CA, USA). Spectra were recorded in a 200–800 nm range, at a 1 nm/s scan rate, and the DCF amounts were monitored by measuring the absorbance intensity at λ = 277 nm. The following molar absorption coefficients (ε) were used, 9800 L·mol^−1^·cm^−1^, to infer the DCF concentration.

### 3.9. FTIR-ATR Spectroscopy Measurements

FTIR-ATR analyses were performed on chitosan film before and after the DCF adsorption. More specifically, the DCF was adsorbed on 0.05 g of chitosan film (Film 2) from a 1.50 × 10^−5^ M solution. FTIR-ATR spectra were recorded in a 600–4000 cm^−1^ range, at a resolution of 4 cm^−1^, using a Fourier Transform Infrared spectrometer 670-IR equipped with an ATR device (Varian Inc., now Agilent Technologies Inc., Santa Clara, CA, USA). Thirty-two scans were summed for each acquisition. The DCF powder was also investigated.

### 3.10. Swelling Ratio Measurements of Chitosan Film with and without DCF

The chitosan films, in the absence and in the presence of DCF, were subject to swelling ratio measurements. The films were cut to a square piece of 0.5 cm × 0.5 cm and swollen in bi-distilled water, at controlled room temperature. Measurements were performed every minute until the equilibrium was attained. The films were blotted with filter paper and weighed. To infer the swelling ratio percentage, Equation (2) was used [10].

(2) $$\mathrm{Swelling}\;\mathrm{ratio}\;\%=\;\frac{W_{s}-W_{d}}{W_{d}}\times 100$$

where W_s_ is the weight of the swollen film at time t and W_d_ is the weight of the dried film (the weight of each film before contact with water).

### 3.11. Thermodynamic Studies

Free energy (ΔG°), entropy (ΔS°), and enthalpy (ΔH°) were calculated for the DCF adsorption on chitosan film at the three selected temperatures: 318, 298, and 278 K [5,23]. More specifically, the ΔG° was inferred by using Equation (3).

ΔG° = −RT ln K_eq_ (3)

in which R is the universal gas constant (8.314 J/mol·K), T is the temperature (K), and K_eq_ represents the equilibrium constant. The ΔH° and ΔS° were determined by combining Equation (4) with Equation (3). Equation (5) was, thus, obtained.

ΔG° = ΔH° − TΔS°(4)

(5) $$\ln\;K_{\mathrm{eq}}\;=\;-\frac{\Delta H{}^{\circ}}{\mathrm{RT}}\;+\;\frac{\Delta S{}^{\circ}}{R}$$

### 3.12. Adsorption Isotherms

The Langmuir, Freundlich, and Temkin isotherms in their linearized forms (Equation (6), Equation (7), and Equation (8), respectively) [5,23] were used to study the bio sorption process of DCF on the presented adsorbents. The assumption of Langmuir model is that the adsorption occurs on homogeneous surfaces with uniformly energetic adsorption sites and monolayer coverage [25]. The interaction between adsorbed molecules is not predicted. Equation (6) reports the Langmuir equation.

(6) $$\frac{C_{e}}{q_{e}}=\frac{1}{K_{L}Q_{0}}+\frac{C_{e}}{Q_{0}}$$

where q_e_ (mg·g^−1^) is the adsorbed amount of DCF at equilibrium, C_e_ is the equilibrium concentration of the DCF (mg·L^−1^) in solution, K_L_ is the Langmuir equilibrium constant (L·mg^−1^), and Q_0_ is the maximum adsorption capacity (mg·g^−1^). The assumption of the Freundlich model is that the surface of the adsorbent is heterogeneous, and the adsorption sites have different energy of adsorption. The energy of adsorption varies as a function of the surface coverage. Equation (7) reports the linear form of this equation.

(7) $$\log\left(q_{e}\right)=\log\left(K_{F}\right)+\frac{1}{n}\log\left(C_{e}\right)$$

where K_F_ (L·mg^−1^) is the Freundlich constant and n is the heterogeneity factor. K_F_ is related to the adsorption capacity, whereas the 1/*n* value indicates if the isotherm is irreversible (1/*n* = 0), favorable (0 < 1/*n* < 1) or unfavorable (1/*n* > 1). Equation (8) was used to describe the Temkin model.

(8) $$q_{e}=B_{1}\ln\left(K_{T}\right)+B_{1}\ln\left(C_{e}\right)$$

The constants B_1_ and K_T_ are inferred directly from the slope and the intercept of Equation (8), respectively. K_T_ represents the equilibrium binding constant (L·mol^−1^) corresponding to the maximum binding energy and B_1_ is related to the heat of adsorption. The model indicates that the heat of adsorption during the adsorption process linearly decreases with the coverage due to adsorbent-adsorbate interactions, and the adsorption process is characterized by a uniform distribution of binding energies.

### 3.13. Adsorption Kinetics

Information about the kinetics of the adsorption process between DCF and chitosan film were inferred by adopting both pseudo-first-order and pseudo-second-order kinetic models [10,20,21]. The following linearized equations for the pseudo-first (Equation (9)) and pseudo-second-order (Equation (10)) models were adopted.

(9) $$\ln\left(q_{e}-q_{t}\right)=\ln\left(q_{e}\right)-K_{1}\times t$$

(10) $$\frac{t}{q_{t}}=\frac{1}{K_{2}q_{e}^{2}}+\frac{1}{q_{e}}\times t$$

where q_e_ and q_t_ represent the adsorption capacities at equilibrium and at time *t*, respectively (mg·g^−1^) and k_1_ (min^−1^) and k_2_ (g·(mg·min)^−1^) are the rate constants of pseudo-first and second order models, respectively [10,20,23].

## 4. Conclusions

Considering the actual problem related to the water decontamination from pollutants, this paper reports high efficiency in the DCF removal, and, thus, of NSAID EPs, from water, by using chitosan film. The bio-sorption process showed how 90% of the DCF can be removed in 2 h, at most. The CH maximum adsorption capacity was also inferred (q_max_ ≈ 10 mg·g^−1^). The sorption process was investigated at several temperature values, which indicates that increasing the temperature, increased the removal of the DCF from water. The thermodynamic parameters were calculated, by observing that the process was spontaneous (ΔG° < 0), endothermic (ΔH° < 0), and occurs with an increase of entropy, which suggests the increase of randomness at the surface of the adsorbent material. By using the isotherm models, the Freundlich and the Temkin equations well fitted the experimental data, which indicates the heterogeneous character of the adsorption process, that happened, from a kinetic point of view, following the pseudo second-order kinetic model. Accordingly, the bio-sorption was DCF and adsorbent amounts-dependent. By increasing the weight of the CH and diluting the DCF solution, the pollutant removal efficiency increased, which affects the adsorption capacities. The presence of electrostatic interaction between DCF and protonated chitosan amino groups were found to be responsible for the affinity between the sorbent and the NSAID. The adsorption was largely affected by the pH values of the DCF solution and by the presence of salts, with the maximum removal observed at pH 5. Under this condition of work, the DCF and chitosan were negatively and positively charged, respectively, and the interaction between the carboxylic group of DCF and amino groups of chitosan were favored. Since the ionic strength affects the affinity between DCF and CH, the use of 0.25 M NaCl could be used for the desorption of the adsorbed DCF, by proposing the 100% reuse of the pollutant and the recycling of the adsorbent for several cycles, which extends the CH lifetime. The high potential of the CHs was highlighted, considering the possibility to induce the solid-state degradation of the adsorbed DCF. Among the proposed approach reported in literature to treat organic molecules, the only use of the light occurred suitable for our purpose. Additionally, the use of CH was also evaluated in the presence of Ketoprofen and an EPs mixture (Ketoprofen and Diclofenac), which shows the high performance of the adsorbent working both under static and dynamic conditions. In fact, the results obtained during in flux measurements open the possibility to use the adsorbent on a large scale for industrial applications.

## Supplementary Materials

The following are available online https://www.mdpi.com/2218-273X/9/10/571/s1. Scheme S1: Chemical structure of DCF, Figure S1: Application of the Weber-Morris model. Figure S2: The percentage of DCF adsorption onto chitosan film in the presence of NaCl at different concentrations. Figure S3: Isotherms of adsorption. Figure S4: Pseudo-first and Pseudo-second order kinetic models as a function of chitosan film sizes. Figure S5: Pseudo-first and Pseudo-second order kinetic models as a function of different DCF concentrations. Figure S6: Percentage of DCF desorption in presence of NaCl, KCl, and MgCl2. Figure S7: Percentage of DCF adsorption/desorption in NaCl and KCl at different concentrations. Figure S8: Camera picture of the experimental setup adopted during the influx experiments and time evolution of the UV-Vis spectrum of DCF solution before and after the flow. Figure S9: UV-Vis spectra of DCF by-products. Figure S10: UV-Vis spectrum of the desorbed DCF by-products. Figure S11: Time evolution of the UV-Vis spectrum of the desorbed DCF by-products in NaCl solution. Figure S13: Time evolution of the UV-Vis spectrum of the Kp and DCF mixture. Table S1: the percentage of DCF adsorption. Table S2: Thermodynamic parameters. Table S3: Isotherm parameters. Table S4: Kinetic parameters. Table S5: Kinetic parameters.
