# Supplementary material for: Chitosan Film as Eco-Friendly and Recyclable Bio-Adsorbent to Remove/Recover Diclofenac, Ketoprofen, and Their Mixture from Wastewater
Source: Biomolecules. 2019 Oct 5;9(10):571. doi: 10.3390/biom9100571 (PMC6843693; doi:10.3390/biom9100571)
Supplement: Supplementary file 1 [file biomolecules-09-00571-s001.pdf]

Electronic Supporting Information

Chitosan film as eco-friendly and recyclable bioadsorbent to remove/recover Diclofenac, Ketoprofen and their mixture from wastewater

Vito Rizzi[a], Fabio Romanazzi[a], Jennifer Gubitosa[b], Paola Fini[b], Roberto Romita[a], Angela Agostiano[a], Andrea Petrella[c] and Pinalysa Cosma[a,b*]

[a]Università degli Studi "Aldo Moro" di Bari, Dip. Chimica, Via Orabona, 4-70126 Bari, Italy;

[b]Consiglio Nazionale delle Ricerche CNR-IPCF, UOS Bari, Via Orabona, 4-70126 Bari, Italy;

[c]Dipartimento di Ingegneria Civile, Ambientale, del Territorio, Edile e Chimica, Politecnico di Bari, via Orabona, 4-70126 Bari, Italy.

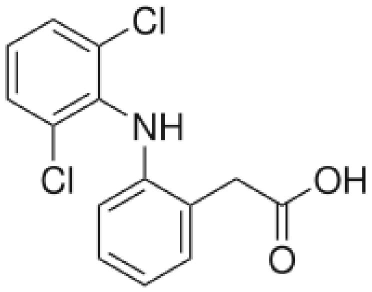

Scheme S1: Chemical structure of DCF.

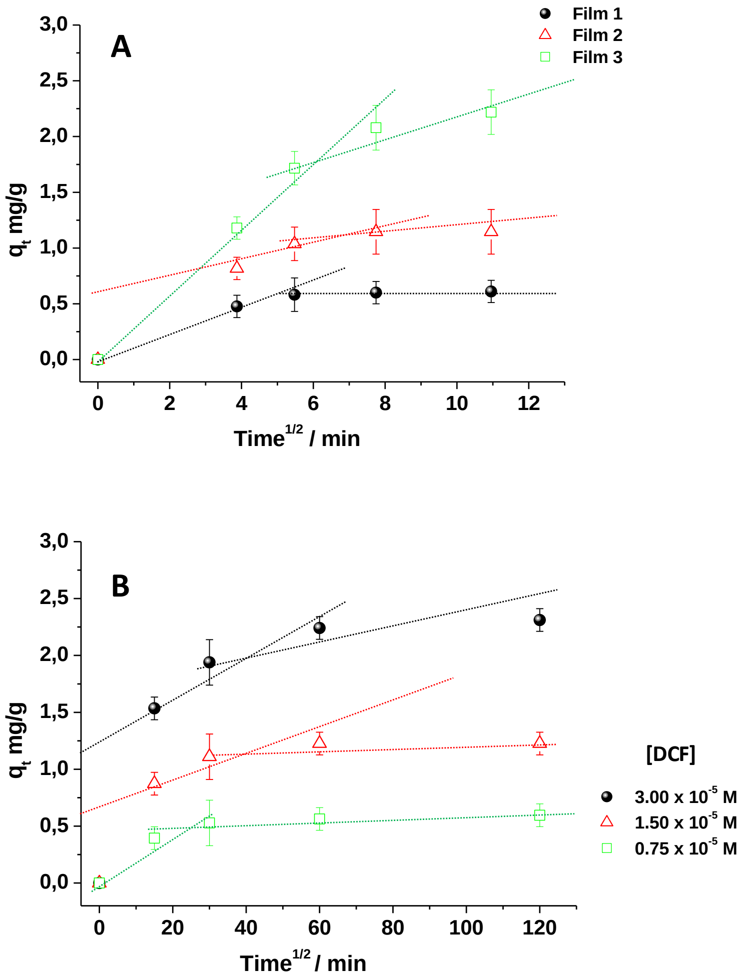

Figure S1: The Weber-Morris model applied to the chitosan adsorption capacities by adopting a DCF solution 1.50×10^{-5} M, at pH 5, and three different chitosan film (having different sizes), Film 1 > 2 > 3 (**A**); and by changing the DCF concentrations, 0.75×10^{-5} M, 1.50×10^{-5} M, 3.00×10^{-5} M, at pH 5, using Film 2 (**B**).

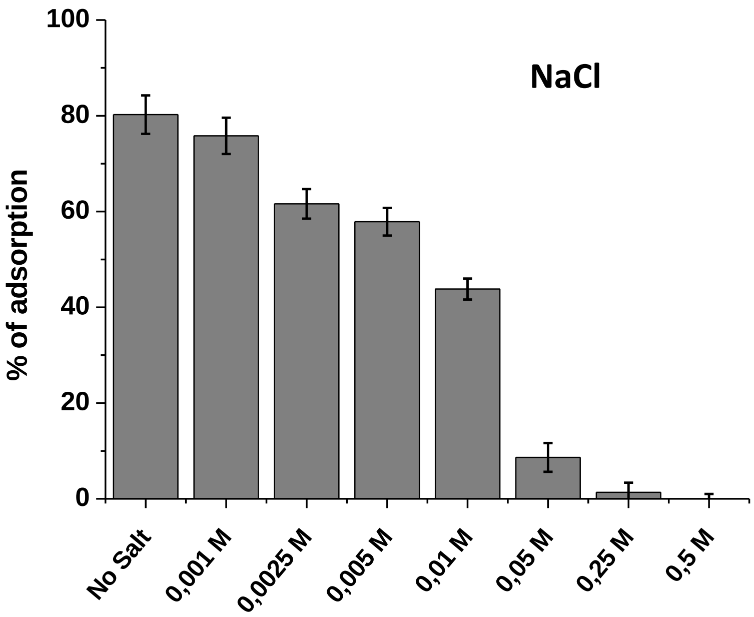

Figure S2: The % of DCF adsorption onto chitosan film (Film 2), at pH 5, (from a DCF solution 1.50×10^{-5} M), in presence of NaCl at different concentrations.

N° of Cycle	% of DCF adsorption
1	81 ± 4
2	86 ± 5
3	89 ± 5
4	85 ± 4
5	78 ± 6
6	56 ± 3
7	66 ± 2
8	71 ± 5
9	60 ± 7
10	56 ± 8

Table S1: The % of DCF adsorption onto chitosan film (Film 2), at pH 5, (from a DCF solution 1.50×10^{-5} M), calculated for 10 consecutive cycles of adsorption by using the same film and by changing after each cycle the DCF solution. The contact time for each cycle was 120 minutes.

ΔH^0 (kJ/mol)	ΔS^0 (J/mol k)	ΔG^0_{278K} (kJ/mol)	ΔG^0_{298K} (kJ/mol)	ΔG^0_{318K} (kJ/mol)
+ 22,62	+ 127,37	-12,78	-15,33	-17,88

Table S2: Thermodynamic parameters refereed to the DCF adsorption onto chitosan film.

LANGMUIR

$$\frac{C_e}{q_e} = \frac{1}{K_L Q_0} + \frac{C_e}{Q_0}$$

FREUNDLICH

$$\log(q_e) = \log(K_F) + \frac{1}{n}\log(C_e)$$

TEMKIN

$$q_e = B_1 ln(K_T) + B_1 ln(C_e)$$

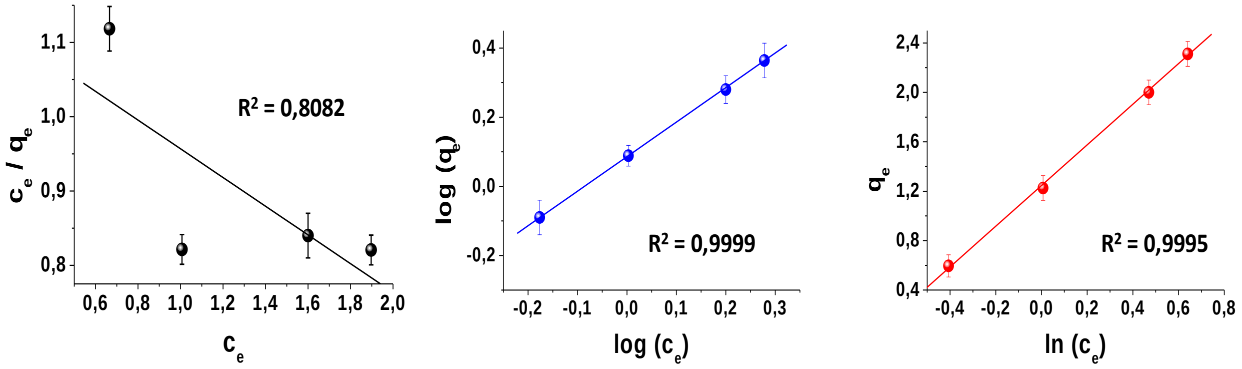

Figure S3: Isotherms of adsorption

Freundlich isotherm model			Temkin isotherm model		
K_F (L×mg^{-1})	n	R^2	K_T(L×mol^{-1})	B_1	R^2
1.20	1.01	0.9999	1,90	1.65	0.9995

Table S3: Isotherm parameters for the adsorption of DCF onto chitosan film.

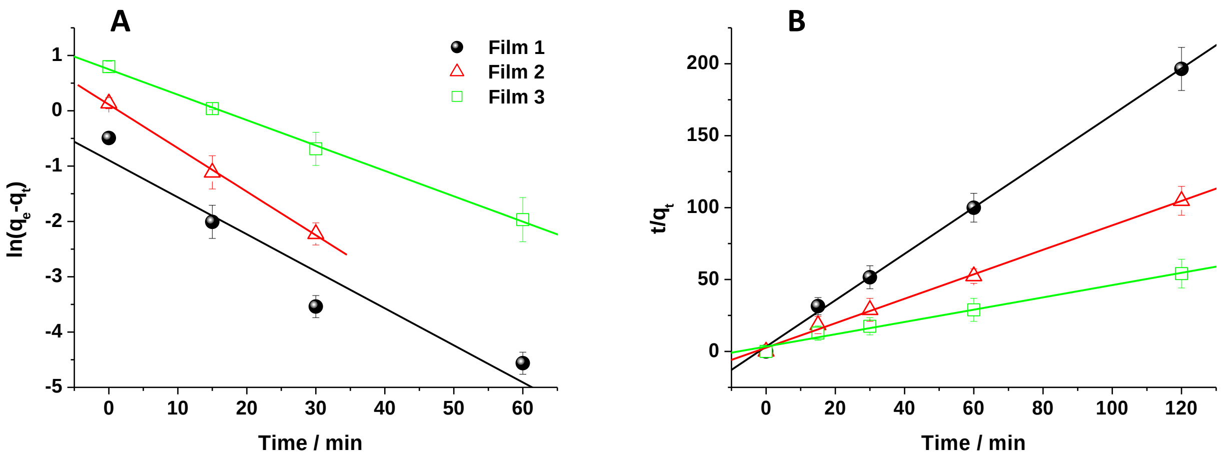

Figure S4: Pseudo-first (**A**) and Pseudo-second (**B**) order kinetic models applied to experimental data. The adsorption capacities are referred to experiments performed by adopting a DCF solution 1.50×10^{-5} M, at pH 5, and three different chitosan film sizes, Film 1 > 2 > 3.

	Pseudo first-order				Pseudo second-order			
**Film**	q_e *exp.*	q_e *calc.*	K_1	R^2	q_e *exp.*	q_e *calc.*	K_2	R^2
**1**	0,61	0,41	0,067	0,9265	0,61	0,62	0,787	0,9989
**2**	1,15	1,02	0,079	0,9989	1,15	1,16	0,277	0,9973
**3**	2,22	2,08	0,046	0,9981	2,22	2,24	0,054	0,9868

Table S4: Kinetic parameters referred to experiments performed by adopting a DCF solution 1.50×10^{-5} M, at pH 5, and three different chitosan film sizes, Film 1 > 2 > 3.

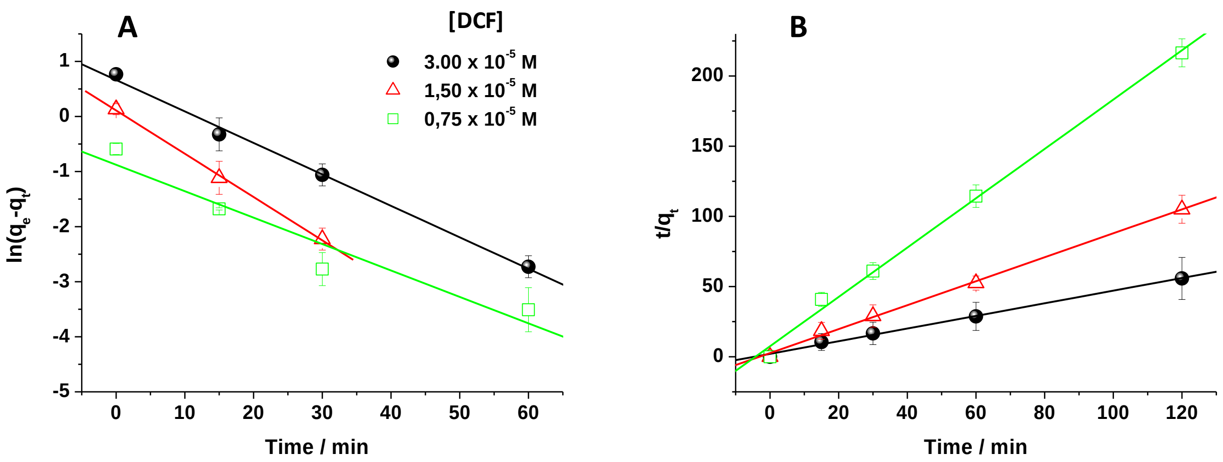

Figure S5: Pseudo-first (**A**) and Pseudo-second (**B**) order kinetic models applied to experimental data. The adsorption capacities are referred to experiments performed by adopting DCF solutions 0.75×10^{-5} M, 1.50×10^{-5} M and 3.00×10^{-5} M, at pH, in presence of Film 2.

	Pseudo first-order				Pseudo second-order			
**DCF Concentration**	q_e *exp.*	q_e *calc.*	K_1	R^2	q_e *exp.*	q_e *calc.*	K_2	R^2
**3.00×10^{-5} M**	2,15	1,94	0,057	0,9955	2,15	2,20	0,098	0,9954
**1.50×10^{-5} M**	1,14	1,02	0,079	0,9989	1,14	1,16	0,280	0,9972
**0.75×10^{-5} M**	0,55	0,42	0,048	0,9271	0,55	0,56	0,411	0,9960

Table S5: Kinetic parameters related to experiments performed by adopting DCF solutions 0.75×10^{-5} M, 1.50×10^{-5} M and 3.00×10^{-5} M, at pH 5, in presence of Film 2.

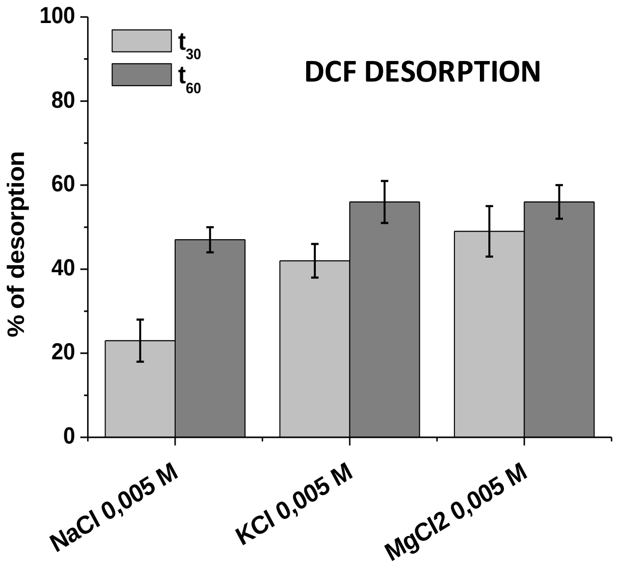

Figure S6: % of DCF desorption in presence of NaCl, KCl and $MgCl_2$, 0.005 M, from CH, after 30 and 60 minutes adopted as contact time.

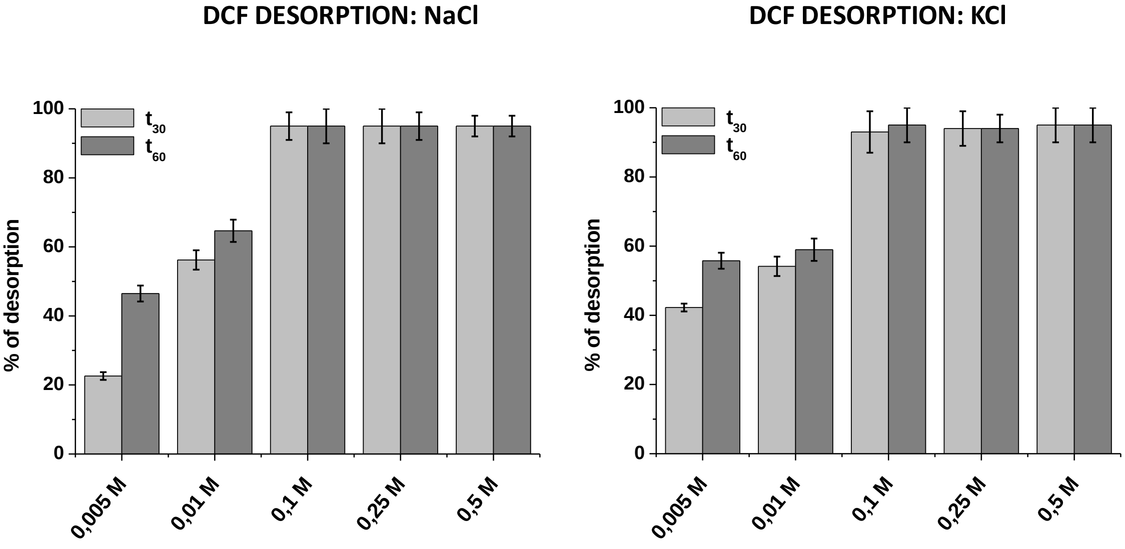

Figure S7: % of DCF adsorption/desorption in NaCl (**A**) and KCl (**B**) at different concentrations, on/from CH at 30 and 60 minutes as contact time.

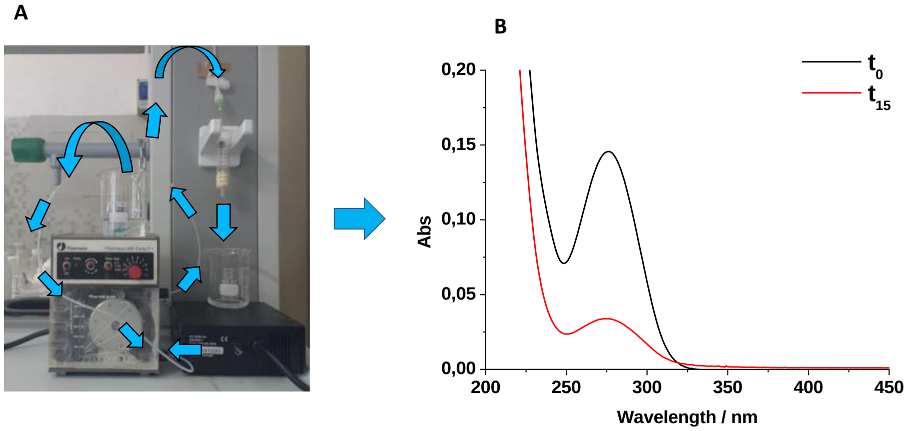

Figure S8: Camera picture of the experimental setup adopted during the *in flux* experiments using CH as adsorbent and DCF as model EP. The arrows in the Figure indicate the direction of the flux (**A**); Time evolution of the UV-Vis spectrum of DCF solution before and after the flow through the column (contact time 15 minutes).

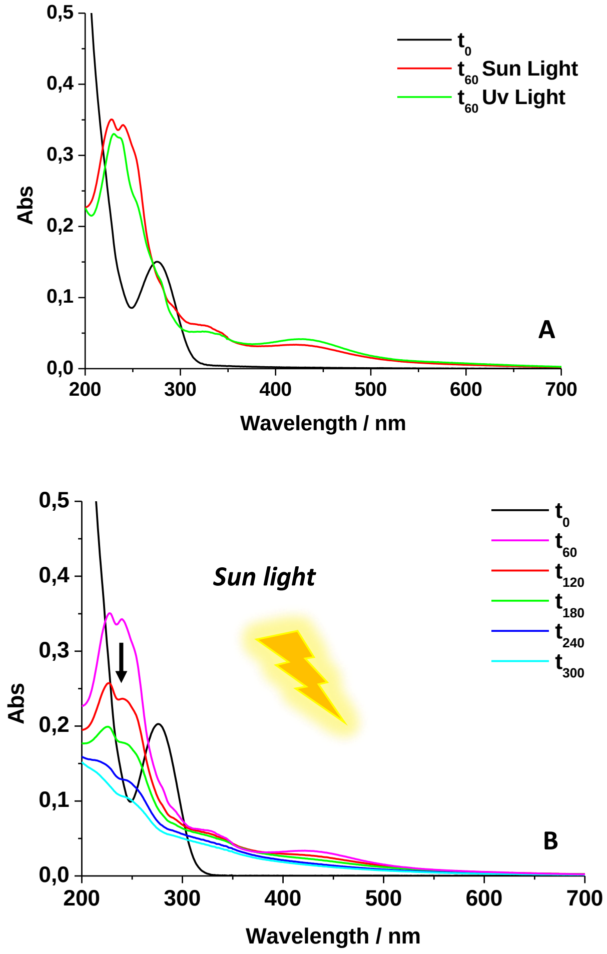

Figure S9: UV-Vis spectra of DCF by-products. The DCF solution was irradiated with UV or Sun light for 60 minutes (**A**); and extending the contact time to 300 minutes (**B**).

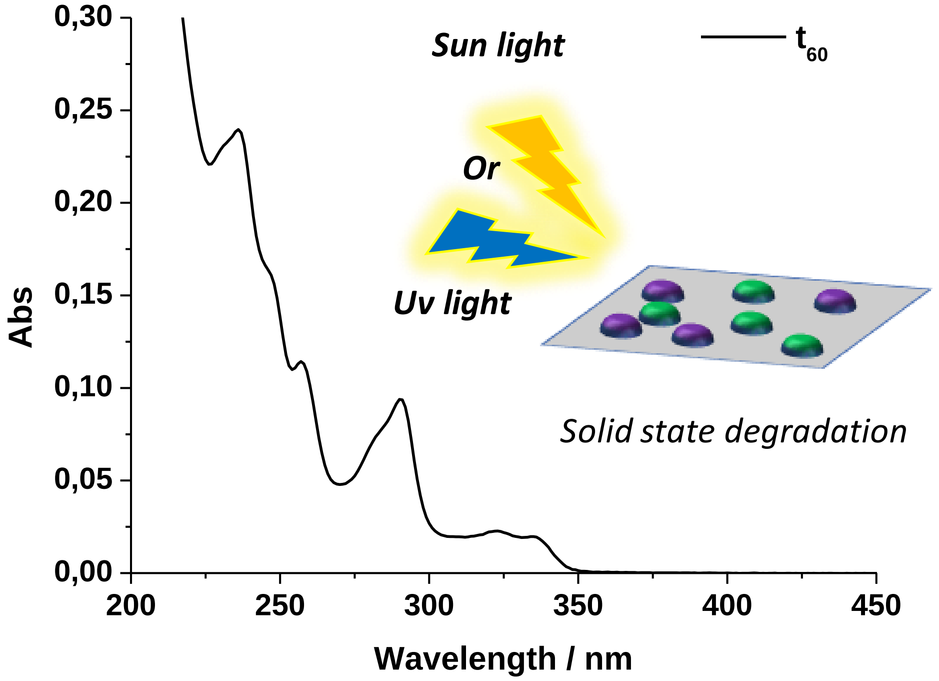

Figure S10: UV-Vis spectrum of the desorbed DCF by-products (in 0.25M NaCl solution), from CH, after 60 minutes of the solid-state irradiation with light. The cartoon depicts the irradiation process transforming the adsorbed DCF molecules (violet spheres) in DCF by-products (green spheres).

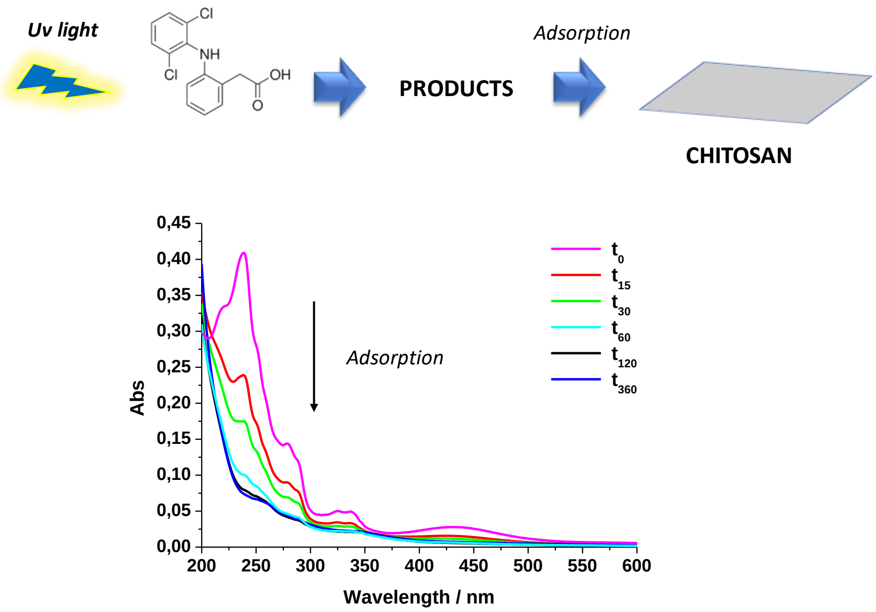

Figure S11: Time evolution of the UV-Vis spectrum of the desorbed (in 0.25 M NaCl solution) DCF by-products, from CH, after 60 minutes of irradiation with light and the subsequently adsorption onto CH at several contact time.

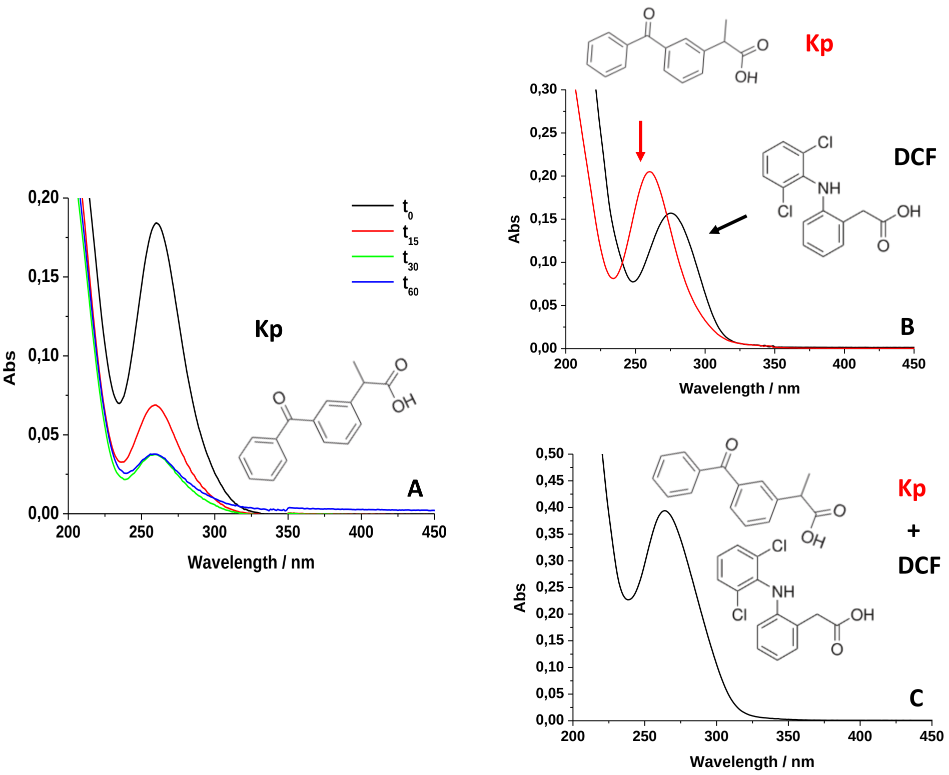

Figure S12: Time evolution of the UV-Vis spectrum of Kp (1.50×10^{-5}M) during the adsorption onto CH (**A**); Comparison between the Kp and DCF UV-Vis spectra (**B**); UV-Vis spectrum of the DCF and Kp mixture (1.50×10^{-5}M × 2) (**C**).

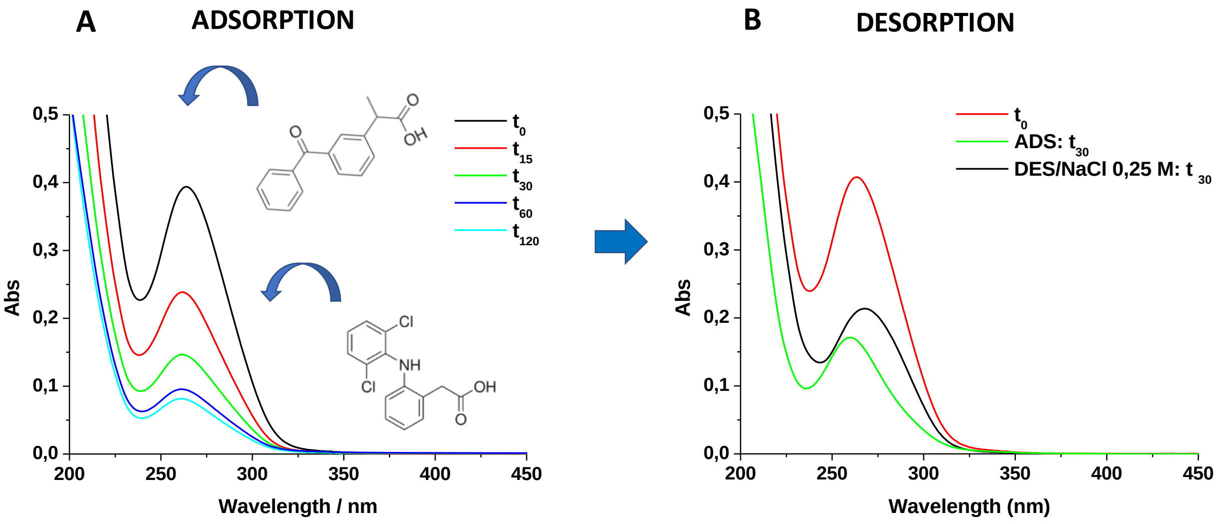

Figure S13: Time evolution of the UV-Vis spectrum of the Kp and DCF mixture (1.50×10^{-5}M × 2) during the adsorption onto CH (**A**); Desorption of the Kp and DCF mixture in NaCl 0.25 M after 30 minutes adopted as contact time. In the Figure the UV-Vis spectrum of the mixture before the adsorption and after the DCF removal in 30 minutes are reported (**B**).
